# Physical and emotional health among nurses in protracted crisis settings in Lebanon and Jordan: A cross-sectional study

**DOI:** 10.1371/journal.pone.0352022

**Published:** 2026-06-23

**Authors:** Nuhad Dumit, Gladys Honein-AbouHaidar, Muntaha K. Gharaibeh, Ibtisam M. Al-Zaru, Reem Hoteit, Imad Bou-Hamad, Patricia Davidson, Nancy Reynolds

**Affiliations:** 1 Hariri School of Nursing, American University of Beirut, Beirut, Lebanon; 2 Global Health Institute, American University of Beirut, Beirut, Lebanon; 3 Faculty of Nursing, Jordan University of Science and Technology, Irbid, Jordan; 4 Department of Business Information and Decision Systems, Suliman S. Olayan School of Business, American University of Beirut, Beirut, Lebanon; 5 International Centre for Future Health Systems, University of New South Wales, Sydney, New South Wales, Australia; 6 School of Nursing, Johns Hopkins University, Baltimore, Maryland, United States of America; University of Petra (UOP), JORDAN

## Abstract

**Objectives:**

Nurses working in crisis-affected and refugee-hosting settings face demanding conditions that may compromise their physical and emotional health. Evidence on the prevalence and determinants of these outcomes in such contexts remains limited. This study assessed nurses’ physical and emotional health in Lebanon and Jordan and identified key associated factors using regression and random forest models.

**Methods:**

A cross-sectional survey was conducted among hospital nurses providing care to Syrian refugees in Lebanon (n = 976; response rate 52%) and Jordan (n = 2,012; response rate 80.5%). Back pain, general weakness and emotional exhaustion outcomes were assessed alongside sociodemographic, work-related, and psychosocial work variables. Logistic, linear regression and random forest models were performed on merged and country-specific datasets.

**Results:**

Nearly six in ten nurses in Lebanon and more than seven in ten in Jordan reported strong general weakness, and over 60% in both countries experienced strong back pain. Emotional exhaustion was prevalent and higher among Jordanian nurses (4.13, SD = 1.39) compared to Lebanese nurses (3.49, SD = 1.70). Age, gender, working hours, and work unit were significant demographic and occupational predictors, while job conflict, self-perceived workload, lack of job preparation, and workload stress emerged as important work environment factors. Nursing resources and teamwork were consistently protective. Random forest models confirmed the relative importance of workload-related factors and highlighted the close association between physical health and emotional exhaustion.

**Conclusion:**

Hospital nurses caring for refugees in Lebanon and Jordan experience high levels of physical and emotional health strains associated with workload, work unit, psychosocial strain, and organizational resources. Findings highlight the need for system-level workforce policies, including workload regulation, staffing optimization, and strengthened organizational support to promote nurse well-being and enhance health system resilience in refugee-hosting and resource-constrained settings.

## Introduction

Globally, there have been significant shifts in population due to geopolitical crises and climate emergencies. The Syrian refugee crisis is considered the largest mass displacement of people in the twenty-first century, with more than 12 million Syrians forced to leave their homes [1]. Of these, over five million have sought refuge in neighboring Middle Eastern countries such as Turkey, Lebanon and Jordan. As of 2025, the number of registered and unregistered Syrian refugees in Lebanon remains around 1.4 million [2], giving the country the highest per capita refugee population in the world [3]. Similarly, Jordan hosts one of the world’s largest refugee populations relative to its size, accommodating more than 1.3 million Syrians, more than 80% reside outside formal refugee camps [2].

The healthcare systems in both countries have been heavily burdened by the Syrian refugee influx. Jordan’s public health system faces increased service demands, rising costs, and widespread affordability barriers [4]. In Lebanon, the influx has generated a marked increase in demand for healthcare services, placing additional strain on an already fragile system [5,6]. It is of note that Lebanon’s highly privatized health system is characterized by greater out-of-pocket expenditures, limited insurance coverage, and high utilization rates, making cost a major barrier to access to healthcare [7]. Besides, the growing prevalence of chronic diseases among refugees in both countries, has placed long-term pressure on health infrastructure and resources extending beyond immediate emergency care needs [8].

It is worth noting that Jordan and Lebanon share similar life expectancy and high unemployment, yet differ markedly in population size and income level: in 2024, Lebanon’s population was about 5.8 million and classified as a lower-middle-income economy, whereas Jordan’s reached 11.8 million and is classified as an upper-middle-income country [9]. These structural differences shape each country’s capacity to absorb and sustain large refugee populations; many Syrian refugee households in Jordan live below the international poverty line and face barriers to essential services [10], while in Lebanon the situation is even more severe, with over half of the host population living below the poverty line and nearly 90% of Syrian refugees requiring humanitarian assistance [11]. Together, these conditions have intensified vulnerabilities and placed additional pressure on already strained health systems in both countries [9,11].

Against this backdrop, resilient health systems in both countries and globally depend on a well-supported health workforce. Nurses play a vital role in delivering high-quality care, yet substantial global shortages persist [12,13]. The World Health Organization (WHO) projects a shortage of about 4.5 million nurses by 2030 [14], with nearly 69% concentrated in the African and Eastern Mediterranean regions [15]. Such workforce gaps contribute to stressful work environments and may undermine care quality and system sustainability [16,17].

In Lebanon, Jordan, and other Eastern Mediterranean countries, these global shortages are compounded by low nurse-to-population ratios and fragile labor markets [15,18]. In Lebanon, economic collapse, currency devaluation, inflation, and political instability have further accelerated nurse migration [19–21]. Together with demographic pressures and ongoing political crises, these factors place additional strain on already overstretched health systems. The COVID-19 pandemic further intensified pre-existing pressures on nurses, adding sustained stress, burnout, and retention challenges to already fragile health systems [22,23].

Evidence indicates that nurses are more likely than other professionals to suffer from physical illnesses, mental distress, and emotional fatigue [24,25]. Healthy work environments are essential for promoting nurses’ well-being and productivity, and workplace characteristics have been linked to nurses’ physical and emotional health [26,27]. Sampson et al. (2020) showed that nurses are twice as likely to suffer from depression, and more than half report poor physical and mental health [28]. Work-related musculoskeletal disorders are also highly prevalent among nurses, with a pooled prevalence of 77.2%, most commonly affecting the lower back, neck, and shoulders [29]. On another note, workload demands, nurse supervisors’ competencies and leadership, staffing adequacy, and the availability of support and teamwork atmosphere are key factors associated with nurses’ resilience and their physical and emotional health in hospitals [27,30].

Studies from Lebanon and Jordan highlight the additional challenges faced by nurses providing care to Syrian refugees. In Jordan, Oweidat et al. (2024) found that poor work quality of life among nurses working in refugee-camp settings was strongly associated with resource constraints, higher turnover, and burnout, undermining continuity and quality of care [31]. Additionally, a qualitative study by Backlund and Olausson (2021) with Jordanian nurses in refugee camps and public hospitals reported that strong empathy for refugees coexisted with limited resources, insecure employment, and a limited scope of practice, all of which reduced nurses’ ability to deliver tailored, high-quality care [32]. In Lebanon, Dumit et al. (2019) explored the impact of the Syrian refugee crisis on nurses and the broader healthcare system and reported burnout, reduced compassionate care, strained interpersonal relationships, and challenges related to resource shortages and shifts in host communities’ use of services [33].

Despite these contributions, the literature on the impact of the Syrian refugee crisis on nurses’ well-being in Lebanon and Jordan remains limited, with few addressing the physical and emotional health of hospital nurses directly involved in refugee care. Given ongoing workforce shortages and increasing workload demands, there is a critical need to examine how these factors affect nurses’ physical and emotional health. Therefore, this study aims to fill this gap by conducting a quantitative investigation of nurses’ experiences in both Lebanon and Jordan. The objectives were to: (a) assess nurses’ physical and emotional health in Lebanon and Jordan within the context of the prolonged Syrian refugee crisis; (b) examine and compare the factors associated with these outcomes using both combined and country-specific analyses; (c) identify the key determinants of nurses’ physical and emotional health through random forest regression on the merged dataset, enabling cross-country comparison.

To complement regression analyses, we applied random forest as a machine learning approach well suited to complex health workforce data. Random forest is a non-parametric ensemble method that can capture non-linear relationships and interactions among predictors while also providing variable-importance rankings that help identify the most influential determinants of an outcome [34,35]. This is particularly relevant in health workforce research, where physical and emotional health are shaped by multiple, interrelated occupational and organizational factors that may not be fully captured by conventional regression alone [36]. In this study, random forest was therefore used as a complementary method to strengthen interpretation of the most relevant predictors of nurses’ physical and emotional health in Lebanon and Jordan.

## Materials and methods

This study represents a substudy of the larger PROfILE project (Perspectives of Registered Nurses on Refugee Healthcare in Lebanon and Jordan) [37], focusing on nurses’ physical and emotional health outcomes and their associated predictors.

### Study design and participants

The PROFILE parent study employed a cross‐sectional, non-experimental design conducted in hospital settings in Lebanon and Jordan [37]. The study population comprised licensed nurses registered with the relevant national health authority. All eligible nurses within participating hospitals were invited to participate. Only those who offered direct health care services to Syrian refugees for a minimum of one year were included. According to the project’s inclusion and exclusion criteria, approximately 3,000 nurses provided health care services to Syrian refugees [37]. Nurses who did not provide direct care, such as academic nurses and supervisors, were excluded. In Lebanon, eligible nurses were identified through the data of Order of Nurses from Lebanon, with support from an Order administrative assistant. In Jordan, eligible nurses were identified through hospital administrative records at participating facilities, in coordination with hospital nursing administrations.

### Data collection

Data collection took place in Jordan and Lebanon from October to December 2019 using a hard copy self-administered questionnaire, accompanied by return-sealed envelopes to maintain confidentiality. Each questionnaire came with a cover letter functioning as a consent document. Nurses were given a two-week period to complete the survey, which was estimated to take between ten and fifteen minutes.

In Lebanon, we distributed 3,000 surveys to registered nurses employed in hospitals and primary healthcare centers serving Syrian refugees. Out of these, we received 1,560 complete questionnaires, and 976 were included in the final analysis (hospital nurses). In Jordan, we distributed 2,500 surveys under similar conditions and received 2,012 completed questionnaires, all of which were included in the analysis. The response rate was 52% in Lebanon and 80.5% in Jordan.

### Ethics approval and consent to participate

This study was approved by the Institutional Review Board (IRB) of the Jordan University of Science and Technology (IRB No. 19/123/2019) and the American University of Beirut (IRB No. NUR.ND.15/SBS-2018–0229).

Written informed consent was obtained from all participants. The study was conducted in accordance with the Declaration of Helsinki principles for research involving human participants.

### Measures

The survey instrument consisted of questions assessing: (1) sociodemographic characteristics; (2) work-related and organizational factors; (3) psychosocial work environment factors; and (4) physical and emotional health outcomes.

#### Hospital nurses’ socio-demographic characteristics.

Socio-demographic characteristics included age (categorical: 20–30, 31–40, 41–50, > 50), gender (binary: male and female), marital status (categorical: single, engaged/married and separated/divorced/widow) and educational attainment (categorical: technical nursing diploma, and university degree (e.g Bachelor’s degree in nursing and Masters in nursing).

#### Work-related and organizational factors.

The following work-related variables were included: shift type (categorical: day, evening, night); number of hours worked per week (categorical: < 42.5, 42.5, > 42.5); working years with Syrian Refugees (categorical: 1–2 year, 3–4 years, ≥ 5 years) and the work unit (categorical).

#### Psychosocial work environment factors.

In addition to these structural factors, a set of psychosocial work environment factors—including self-perceived workload, work stressors, teamwork, leadership, nursing resources, and resilience—were assessed to capture the psychological and social dimensions of nurses’ professional experience.

##### Self-perceived workload:

The NASA Task Load Index (NASA-TLX) scale [38] was used to assess participants’ self-perceived workload across six domains: mental, physical, temporal, performance, effort, and frustration demand. Each domain was rated from 0 (lowest) to 100 (highest).

An overall workload score was calculated by averaging the six domain scores, resulting in a composite score ranging from 0 to 100, where higher scores indicate greater perceived workload. The overall workload scale demonstrated acceptable internal consistency, with a Cronbach alpha of 0.72 reported by Hoonakker et al. (2011) and 0.76 in the present study.

##### Work stressors:

The Health Professions Stress Inventory (HPSI) is a widely used questionnaire that assesses the stress levels of professionals in the health care field [39]. On a 5-point Likert scale (0 = never, 1 = seldom, 2 = sometimes, 3 = often, 4 = very often), 14 questions from the HPSI were selected to measure work stressors. Work stressors were categorized into three domains: workload stress (4 items), lack of job preparation (7 items), and job conflict (3 items). For each domain, an average score was computed across the relevant items to obtain a mean value representing the level of that specific work stressor. Cronbach alpha coefficients for the three domains were 0.83, 0.87 and 0.71 respectively, indicating acceptable to good internal consistency.

##### Nursing resources and leadership:

A total of nine items from the Practice Environment Scale of the Nursing Work Index (PES-NWI) [40] were used to assess perceptions of leadership and staffing resource adequacy. Of these, five items measured “Nurse manager ability, leadership, and support of care workers” (Cronbach alpha = 0.84) and four items measured “Staffing and resources adequacy” (Cronbach alpha = 0.74). Responses were measured on a four-point Likert scale (1 = strongly disagree; 2 = somewhat disagree; 3 = somewhat agree; 4 = strongly agree). For each subscale, an average score was computed. In the present study, Cronbach’s alpha coefficients for nursing resources and leadership subscales were 0.87 and 0.85, respectively.

##### Teamwork:

Perceived teamwork and collaboration among healthcare professionals were assessed using the Teamwork subscale of the validated Safety Attitude Questionnaire (SAQ) [41]. This subscale consists of six items, each rated on a four-point Likert scale (1 = strongly disagree; 2 = somewhat disagree; 3 = somewhat agree; 4 = strongly agree). An average score was computed across the six items to represent overall teamwork quality. In the present study, the “Teamwork” subscale’s Cronbach’s alpha coefficient was 0.803.

##### Resilience:

Resilience was assessed using the 14-item Resilience Scale (RS-14) [42,43]. Items were rated on a seven-point Likert scale ranging from 1 (strongly disagree) to 7 (strongly agree). An average score was computed across the 14 items to represent the overall level of resilience. The instrument’s internal reliability has been reported to range between Cronbach’s alpha 0.67–0.84. The Resilience Scale (RS-14) was validated in Arabic [44]. In the present study, the RS-14 showed excellent internal consistency, with a Cronbach’s alpha coefficient of 0.947.

#### Outcome variables.

##### Nursing outcomes:

*Physical health*

Self-reported back pain and general weakness were the two physical health outcomes evaluated using the Swiss Health Survey [45]. On a 3-point Likert scale, the occurrence of these outcomes in the four weeks leading up to the survey (0 = not at all, 1 = a little bit, 2 = strongly) was assessed.

*Emotional health*

The Maslach Burnout Inventory was used to assess nurses’ self-reported emotional exhaustion (MBI). Its nine scale items, which reflect nurses’ feelings of exhaustion from work, were rated on a seven-point Likert scale (ranging from 0 = never to 6 = daily) [46]. All sub-scales were substantively correlated (0.60 < r < 0.76), demonstrating congruent validity [47]. In our study, the emotional exhaustion scale had a Cronbach’s alpha coefficient of 0.87.

For analytical purposes, physical health outcomes were later dichotomized to distinguish nurses reporting strong symptoms from those reporting no or mild symptoms. Given the high proportion of nurses reporting symptoms “strongly,” this approach enabled clearer identification of nurses experiencing more severe physical symptoms and supported risk-focused analysis using logistic regression.

### Inclusivity in global research

Additional information regarding the ethical, cultural, and scientific considerations specific to inclusivity in global research is included in the Supporting Information (S1 Checklist).

### Decision trees and random forests

Decision trees are data-driven techniques for classifying or predicting outcomes, valued for their simple structure and interpretability [48,49]. Classification trees handle categorical outcomes, while regression trees address numerical ones, splitting data into increasingly homogeneous groups based on predictor conditions. However, decision trees are highly sensitive to noise, leading to the development of ensemble methods such as random forests [50]. This approach combines multiple trees built from bootstrap samples, using random subsets of predictors at each split, and aggregates results to improve accuracy. A key strength of random forests is its ability to generate variable importance measures, such as permutation importance, which assess how much prediction error increases when a predictor’s values are randomly permuted [51]. While these models are useful for identifying patterns and ranking predictors according to their relative predictive importance, they do not establish causal relationships.

### Data analysis

Descriptive statistics were used to summarize demographic characteristics and other study variables. For continuous variables, results were expressed as means and standard deviations (SD), and for categorical variables, as frequencies and percentages. Physical health (back pain and general weakness) and emotional health (emotional exhaustion) were the dependent variables in this study. The physical health variables were categorical, while the emotional health variable was numerical. For each outcome, variable importance measures were generated using RF to determine the relevance of each predictor. The random forest was constructed with 2000 trees. To capture nurses with self-reported compromised health, we dichotomized physical health outcomes: back pain and general weakness: 0 = not at all and a little bit, 1 = strongly.

Ordinary least squares (OLS) regression was performed to study the associations between emotional health and independent variables. The constant-variance assumption (homoscedasticity) was, however, violated. To correct heteroscedasticity, the robust standard errors method was used. We checked for multicollinearity among the factors using the variance inflation factor (VIF) and no major multicollinearity concerns have been detected. Furthermore, to analyze the strength and direction of effect of the independent variables on physical health, logistic regression was used where adjusted Odds ratios (AORs), and 95% confidence intervals were estimated for each independent variable. Analysis was done using R language (version 4.3.3). Statistical significance was set at p-value <0.05.

No imputation procedures were applied. Given the low proportion of missing data across variables and the large sample size, complete case analysis was considered appropriate. Analyses were conducted using available observations for each variable, and the extent of missing data is presented in the descriptive tables.

## Results

### Sociodemographic characteristics

Table 1 presents a summary of the sociodemographic characteristics of the study participants. There were significant differences between Lebanon and Jordan across all sociodemographic variables.

**Table 1:** Socio-demographic characteristics of hospital nurses by country (N = 2988).

Variable	Lebanon n = 976 n (%)	Jordan n = 2012 n (%)
**Age (years)**		
20-30	468 (48.0)	474 (23.6)
31-40	396 (40.6)	1302 (64.7)
41-50	90 (9.2)	216 (10.7)
>50	19 (1.9)	20 (1.0)
*Missing*	3 (0.3)	
**Gender**		
Men	240 (24.6)	670 (33.3)
Women	677 (69.4)	1342 (66.7)
*Missing*		
**Marital status**		
Single	393 (40.3)	349 (17.3)
Engaged/married	542 (55.5)	1537 (76.4)
Separated/divorced/widowed	29 (3.0)	126 (6.3)
*Missing*	12 (1.2)	0 (0)
**Education**		
Technical nursing diploma (BT, TS, LT)	523 (53.6)	295 (14.7)
University degree	448 (45.9)	1717 (85.0)
*Missing*	5 (0.5)	0 (0)

In Lebanon, about half of the nurses (48.0%) were between the ages of 20 and 30, whereas in Jordan this age group accounted for 23.6% of nurses. Both countries had a similar gender distribution, with women accounting for more than two-thirds of nurses. In Lebanon, 40.3% of nurses were single, while in Jordan 76.4% were engaged or married. Approximately half of Lebanon’s nurses (53.6%) received a Technical Nursing Diploma, whereas the majority in Jordan (85.0%) has a university degree.

Overall, the two countries differed markedly in age distribution, marital status, and education level, while maintaining a predominantly female nursing workforce in both settings.

### Work-related and psychosocial work environment factors

The work-related and psychosocial characteristics of hospital nurses are summarized in Table 2. In Lebanon, most nurses worked either day shifts (45.5%) or rotating shifts (41.5%). In Jordan, 36.7% of nurses worked day shifts, while 54.8% worked rotating shifts. More than one third of the surveyed Lebanese nurses (38.7%) reported working more than 42.5 hours per week, compared to 33.2% of Jordanian nurses. Approximately 43.0% of hospital nurses in Lebanon and 68.0% in Jordan have spent at least five years serving the Syrian refugees. Most nurses in Lebanon and Jordan worked in various work units, including the medical-surgical area/burns (21.2%), intensive care unit, pediatrics/NICU/PICU, obstetrics/gynecology, emergency departments.

**Table 2:** Work-related and psychosocial work environment characteristics of hospital nurses by country (N = 2,988).

	Lebanon n = 976 n (%)	Jordan n = 2012 n (%)
**Work characteristics**		
**Shift**		
Day	444 (45.5)	738 (36.7)
Evening	52 (5.3)	107 (5.3)
Night	71 (7.3)	65 (3.2)
Rotating shifts	405 (41.5)	1102 (54.8)
*Missing*	4 (0.4)	0 (0)
**Work Unit**		
Medical-surgical area/burns	207 (21.2)	776 (38.6)
Intensive care unit	176 (18.0)	178 (8.8)
Pediatrics/NICU/PICU	157 (16.1)	223 (11.1)
Obstetrics/gynecology	92 (9.4)	201 (10.0)
Renal dialysis unit	39 (4.0)	48 (2.4)
Operating room	41 (4.2)	115 (5.7)
Emergency	109 (11.2)	291 (14.5)
Oncology	14 (1.4)	0 (0)
Cardiology	24 (2.5)	0 (0)
Administration	5 (0.5)	0 (0)
Ambulatory	10 (1.0)	180 (8.9)
Mental health	11 (1.1)	0 (0)
Multiple units	88 (9.0)	0 (0)
*Missing*	3 (0.3)	0 (0)
**Number of hours worked per week**		
<42.5 hours	292 (29.9)	853 (42.4)
42.5 hours	293 (30.0)	492 (24.5)
>42.5 hours	378 (38.7)	667 (33.2)
*Missing*	13 (1.3)	
**Working years with Syrian refugees**		
1-2 years	248 (25.4)	116 (5.8)
3-4 years	291 (29.8)	535 (26.6)
≥5 years	414 (42.4)	1316 (67.6)
*Missing*	23 (2.4)	0 (0)
**Psychosocial work environment factors**	**Mean (SD)**	**Mean (SD)**
**Self-perceived workload (0–100)**	77.0 (16.5)	84.2 (12.4)
**Work stressors (1–5) Scale**		
Workload stress	2.4 (0.8)	2.6 (0.7)
Lack of Job preparation	1.3 (0.8)	1.3 (0.8)
Job conflict	2.2 (0.9)	2.4 (0.7)
**Nursing resources (1–4)**	2.4 (0.7)	2.1 (0.6)
**Leadership (1–4)**	3.0 (0.7)	2.3 (0.6)
**Teamwork (1–5)**	3.6 (0.7)	3.4 (0.7)
**Nursing resilience (1–7)**	5.8 (0.9)	5.4 (1.0)

Jordanian nurses had a higher mean score (84.2) on the self-perceived workload scale (0–100) than Lebanese nurses (77.0). Work stressors were slightly more prevalent among Jordanian nurses. Jordanian nurses had a higher average workload score (2.6) than Lebanese nurses (2.4). Jordanian nurses showed higher mean scores for job conflict (2.4) than Lebanese nurses (2.2). Both groups reported similar scores for lack of job preparation (mean score of 1.3). Lebanese nurses had higher mean scores on nursing resources, leadership, teamwork, and resilience than Jordanian nurses (Table 2).

Moreover, 58.8% of the participants in Lebanon and 71.8% in Jordan reported strong general weakness, while 61.3% and 63.2% respectively reported strong back pain.

In summary, nurses in Jordan reported higher workload and stress levels, while Lebanese nurses reported more favorable scores for organizational resources, leadership, teamwork, and resilience.

### Associations between sociodemographic/work factors and physical health

Logistic regression analysis for physical health showed that strong general weakness and strong back pain were significantly associated with various sociodemographic and work environment factors.

### Strong back pain

#### Merged model*.*

Strong back pain was significantly associated with several sociodemographic and work-related factors (Table 3). Compared to nurses in Lebanon, those in Jordan had 24% lower odds of reporting strong back pain (AOR = 0.76, 95% CI: 0.59–0.99; p = 0.041). Age was also a strong predictor: nurses aged 31–40 had 1.3 times higher odds (AOR = 1.32, 95% CI: 1.06–1.66; p = 0.015), and those aged ≥41 had nearly twice the odds of reporting strong back pain (AOR = 1.98, 95% CI: 1.39–2.83; p < 0.001) compared with nurses aged 20–30. Female nurses had 1.3 times higher odds than males (AOR = 1.27, 95% CI: 1.05–1.55; p = 0.016).

**Table 3:** Adjusted logistic regression of factors associated with strong back pain among hospital nurses in Lebanon and Jordan.

Variable	A-OR	95% CI	p-value
**Country** (Ref Lebanon)	0.76	[0.59, 0.99]	0.041*
**Age (years)**			
20- 30 (Ref)			
31- 40	1.32	[1.05, 1.66]	0.015*
≥41	1.98	[1.39, 2.83]	0.000*
**Gender**			
Male (Ref)			
Female	1.27	[1.05, 1.55]	0.016*
**Marital status**			
Single (Ref)			
Engaged/married	1.21	[0.96, 1.51]	0.108
Separated/divorced/widowed	1.30	[0.85, 2.00]	0.223
**Work unit**			
Medical-surgical area/burns (Ref)			
Intensive care unit	0.83	[0.62, 1.12]	0.221
Pediatrics/NICU/PICU	1.44	[1.07, 1.96]	0.018*
Obstetrics/gynecology	1.47	[1.05, 2.07]	0.025*
Renal dialysis unit	0.71	[0.43, 1.20]	0.199
Operating room	0.74	[0.51, 1.08]	0.119
Emergency	1.01	[0.78, 1.32]	0.918
Oncology	0.38	[0.10, 1.46]	0.148
Cardiology	0.72	[0.25, 2.21]	0.543
Administration	0.97	[0.09, 21.41]	0.983
Ambulatory	0.97	[0.67, 1.42]	0.868
Mental health	0.43	[0.11, 1.80]	0.216
Multiple units	0.95	[0.48, 1.91]	0.871
**Shift**			
Day (Ref)			
Evening	0.62	[0.42, 0.91]	0.014*
Night	0.54	[0.34, 0.84]	0.006*
Rotating shifts	0.89	[0.72, 1.08]	0.245
**Number of hours worked per week**			
<42.5 hours (Ref)			
42.5 hours	1.62	[1.29, 2.02]	0.000*
>42.5 hours	2.45	[1.99, 3.00]	0.000*
**Working years with Syrian refugees**			
1-2 years (Ref)			
3-4 years	1.02	[0.74, 1.40]	0.902
≥5 years	1.35	[0.99, 1.83]	0.060
**Self-perceived workload**	1.01	[1.00, 1.02]	0.002*
**Work stressors**			
Workload stress	1.15	[1.01, 1.30]	0.036*
Job conflict	1.23	[1.09, 1.39]	0.001*
**Nursing resources**	1.06	[0.91, 1.24]	0.425
**Nursing resilience**	1.20	[1.09, 1.31]	0.000*
**Leadership**	0.92	[0.78, 1.08]	0.313
**Teamwork**	0.86	[0.75, 0.97]	0.017*

Nurses working in pediatrics/neonatal and pediatric intensive care unit (NICU/PICU) and obstetrics/gynecology units had significantly greater odds of experiencing strong back pain than those in medical-surgical areas (AOR = 1.44, 95% CI: 1.07–1.96; p = 0.018 and AOR = 1.47, 95% CI: 1.05–2.07; p = 0.025, respectively). Conversely, nurses working evening or night shifts had lower odds of back pain compared with day-shift nurses (Evening AOR = 0.62, 95% CI: 0.42–0.91; p = 0.014; Night AOR = 0.54, 95% CI: 0.34–0.84; p = 0.006).

Weekly working hours were also strongly associated with back pain. Compared to those working <42.5 hours per week, nurses working 42.5 hours (AOR = 1.62, 95% CI: 1.29–2.02; p < 0.001) and >42.5 hours (AOR = 2.45, 95% CI: 1.99–3.00; p < 0.001) had significantly greater odds of strong back pain. Similarly, higher self-perceived workload scores were linked to greater odds (AOR = 1.01, 95% CI: 1.00–1.02; p = 0.002).

Both workload stress (AOR = 1.15, 95% CI: 1.01–1.30; p = 0.036) and job conflict (AOR = 1.23, 95% CI: 1.09–1.39; p = 0.001) were significantly associated with increased odds of strong back pain. Furthermore, higher resilience scores were associated with greater odds (AOR = 1.20, 95% CI: 1.09–1.31; p < 0.001), whereas better teamwork was protective (AOR = 0.86, 95% CI: 0.75–0.97; p = 0.017).

#### Country-specific context*.*

At the country level, several notable differences were observed between Lebanon and Jordan (S2 Table). In Jordan, age and workload-related factors showed strong associations with strong back pain. Compared with nurses aged 20–30, those aged 31–40 had 1.6 times higher odds (AOR = 1.58, 95% CI: 1.21–2.06; p = 0.001), and those aged ≥41 had threefold higher odds (AOR = 3.05, 95% CI: 1.95–4.79; p < 0.001). Long working hours were also significant: nurses working 42.5 hours (AOR = 1.95; p < 0.001) and >42.5 hours (AOR = 3.00; p < 0.001) had markedly higher odds of strong back pain. Work unit type was another key predictor—nurses in pediatrics/NICU/PICU (AOR = 1.58; p = 0.011) and obstetrics/gynecology (AOR = 1.53; p = 0.031) had greater odds, while those in the operating room had lower odds (AOR = 0.60; p = 0.018). Workload stress also emerged as significant (AOR = 1.21; p = 0.016).

In Lebanon, gender and work stressors were more prominent. Female nurses were nearly twice as likely to report strong back pain as males (AOR = 1.81; p = 0.006). Work stressors, particularly lack of job preparation (AOR = 1.46; p = 0.012) and job conflict (AOR = 1.37; p = 0.025), were significantly associated with higher odds of back pain. Additionally, better nursing resources were protective (AOR = 0.64; p = 0.011), with each one unit increase in nursing resources associated with a 36% decrease in the odds of strong back pain.

Overall, strong back pain was primarily associated with older age, female gender, long working hours, high workload, and job conflict, with country-specific differences in the prominence of stressors and organizational resources.

### Strong general weakness

#### Merged model.

Strong general weakness was associated with multiple sociodemographic and occupational factors (Table 4). Female nurses had significantly higher odds of reporting strong general weakness compared with males (AOR = 1.29, 95% CI: 1.05–1.58; p = 0.014). Marital status was also significant, with engaged or married nurses having higher odds than single nurses (AOR = 1.32, 95% CI: 1.04–1.67; p = 0.023). Nurses working in pediatrics/NICU/PICU and obstetrics/gynecology units reported greater odds of strong weakness compared with those in medical-surgical areas (AOR = 1.72, 95% CI: 1.25–2.40; p = 0.001 and AOR = 1.67, 95% CI: 1.18–2.41; p = 0.005, respectively).

**Table 4:** Adjusted logistic regression of factors associated with strong general weakness among hospital nurses in Lebanon and Jordan.

Variable	A-OR	95% CI	p-value
**Country** (Ref Lebanon)	1.14	[0.86, 1.49]	0.359
**Age (years)**			
20- 30 (Ref)			
31- 40	1.21	[0.95, 1.53]	0.124
≥41	1.21	[0.85, 1.73]	0.300
**Gender**			
Male (Ref)			
Female	1.29	[1.05, 1.58]	0.014*
**Marital status**			
Single (Ref)			
Engaged/married	1.32	[1.04, 1.67]	0.023*
Separated/divorced/widowed	1.49	[0.96, 2.34]	0.082
**Education**			
Technical nursing diploma (BT, TS, LT) (Ref)			
University degree	0.87	[0.69, 1.11]	0.264
**work unit**			
Medical-surgical area/burns (Ref)			
Intensive care unit	1.24	[0.91, 1.71]	0.182
Pediatrics/NICU/PICU	1.72	[1.25, 2.39]	0.001*
Obstetrics/gynecology	1.67	[1.18, 2.41]	0.005*
Renal dialysis unit	0.85	[0.5, 1.44]	0.532
Operating room	0.71	[0.48, 1.05]	0.084
Emergency	1.14	[0.86, 1.52]	0.367
Oncology	0.39	[0.09, 1.52]	0.181
Cardiology	0.56	[0.19, 1.61]	0.273
Administration	0.41	[0.02, 4.42]	0.474
Ambulatory	0.87	[0.59, 1.27]	0.455
Mental health	0.28	[0.07, 1.04]	0.061
Multiple units	0.95	[0.48, 1.89]	0.875
**Shift**			
Day (Ref)			
Evening	0.83	[0.55, 1.26]	0.376
Night	0.57	[0.36, 0.9]	0.015*
Rotating shifts	0.93	[0.75, 1.15]	0.508
**Number of hours worked per week**			
<42.5 hours (Ref)			
42.5 hours	1.45	[1.15, 1.84]	0.002*
>42.5 hours	1.78	[1.43, 2.22]	0.000*
**Working years with Syrian refugees**			
1-2 years (Ref)			
3-4 years	0.99	[0.71, 1.36]	0.936
≥5 years	1.24	[0.9, 1.71]	0.183
**Self-perceived workload**	1.02	[1.01, 1.02]	0.000*
**Work stressors**			
Workload stress	1.23	[1.08, 1.41]	0.002*
Lack of job preparation	0.92	[0.81, 1.05]	0.216
Job conflict	1.38	[1.21, 1.57]	0.000*
**Nursing resources**	0.91	[1.08, 1.41]	0.266
**Leadership**	0.88	[0.81, 1.05]	0.131
**Teamwork**	0.87	[1.21, 1.57]	0.046*

Shift type and working hours were also significant predictors. Nurses working night shifts had lower odds of weakness (AOR = 0.57, 95% CI: 0.36–0.90; p = 0.015) compared with day-shift nurses, while those working 42.5 hours (AOR = 1.45, 95% CI: 1.15–1.84; p = 0.002) and more than 42.5 hours per week (AOR = 1.78, 95% CI: 1.43–2.22; p < 0.001) were significantly more likely to report strong weakness.

Higher self-perceived workload (AOR = 1.02, 95% CI: 1.01–1.02; p < 0.001), workload stress (AOR = 1.23, 95% CI: 1.08–1.41; p = 0.002), and job conflict (AOR = 1.38, 95% CI: 1.21–1.57; p < 0.001) were all significantly associated with higher odds of strong general weakness, while teamwork was slightly protective (AOR = 0.87, 95% CI: 0.76–1.00; p = 0.046).

#### Country-specific context*.*

At the country level, several notable differences were observed between Lebanon and Jordan (S1 Table). In Jordan, long working hours and workload-related factors were strongly associated with strong general weakness. Compared with nurses working less than 42.5 hours per week, those working 42.5 hours had 1.7 times higher odds (AOR = 1.74, 95% CI: 1.32–2.29; p < 0.001), and those working more than 42.5 hours had 2.3 times higher odds (AOR = 2.26, 95% CI: 1.74–2.95; p < 0.001). Nurses working in pediatrics/NICU/PICU units had 1.6 times higher odds of reporting strong general weakness (AOR = 1.56, 95% CI: 1.06–2.28; p = 0.023), while those in the operating room had lower odds (AOR = 0.64, 95% CI: 0.41–0.98; p = 0.040). Each one-unit increase in self-perceived workload score was associated with a 2% increase in the odds of general weakness (AOR = 1.02, 95% CI: 1.01–1.03; p < 0.001), and job conflict remained a strong predictor (AOR = 1.26, 95% CI: 1.08–1.46; p = 0.003).

In Lebanon, sociodemographic and psychosocial factors were more prominent in describing weaknesses. Female nurses had nearly twice the odds of reporting strong general weakness compared to males (AOR = 1.80, 95% CI: 1.17–2.77; p = 0.008), whereas older nurses (≥41 years) had lower odds compared to those aged 20–30 years (AOR = 0.49, 95% CI: 0.25–0.93; p = 0.029). Job conflict was a significant predictor (AOR = 1.57, 95% CI: 1.18–2.08; p = 0.002), while better nursing resources (AOR = 0.62, 95% CI: 0.44–0.89; p = 0.009) and teamwork (AOR = 0.63, 95% CI: 0.44–0.90; p = 0.011) were protective against strong general weakness.

In summary, strong general weakness was associated with long working hours, workload-related stress, and job conflict, while teamwork and nursing resources demonstrated protective effects, particularly in Lebanon.

### Emotional exhaustion

#### Merged model.

In the merged analysis, several sociodemographic and work environment factors were significantly associated with emotional exhaustion (Table 5). Compared to Lebanese nurses, Jordanian nurses reported higher emotional exhaustion scores (β = 0.22, 95% CI: [0.06, 0.39]; p = 0.008). Nurses aged 31–40 years reported slightly lower exhaustion than those aged 20–30 (β = −0.13, 95% CI: [−0.27, 0.00]; p = 0.045). Among marital status categories, those separated, divorced, or widowed had higher exhaustion scores (β = 0.26, 95% CI: [0.01, 0.51]; p = 0.040).

**Table 5:** Adjusted linear regression of factors associated with emotional exhaustion among hospital nurses in Lebanon and Jordan.

Variable	Estimate	S.E.	95% CI	p-value
**Country** (Ref Lebanon)	0.22	0.08	[0.06, 0.39]	0.008*
**Age (years)**				
20- 30 (Ref)				
31- 40	−0.13	0.07	[-0.27, 0.00]	0.045*
≥41	−0.10	0.10	[-0.3, 0.11]	0.360
**Gender**				
Male (Ref)				
Female	−0.07	0.06	[-0.19, 0.05]	0.246
**Marital status**				
Single (Ref)				
Engaged/married	0.05	0.07	[-0.09, 0.19]	0.471
Separated/divorced/widowed	0.26	0.13	[0.01, 0.51]	0.040*
**Education**				
Technical nursing diploma (BT, TS, LT) (Ref)				
University degree	−0.07	0.07	[-0.21, 0.07]	0.322
**Work unit**				
Medical-surgical area/burns (Ref)				
Intensive care unit	0.25	0.09	[0.08, 0.43]	0.004*
Pediatrics/NICU/PICU	0.29	0.09	[0.11, 0.47]	0.002*
Obstetrics/gynecology	0.15	0.10	[-0.04, 0.34]	0.123
Renal dialysis unit	0.23	0.16	[-0.09, 0.54]	0.156
Operating room	0.00	0.14	[-0.27, 0.27]	0.984
Emergency	0.18	0.08	[0.02, 0.33]	0.026*
Oncology	0.09	0.39	[-0.68, 0.86]	0.813
Cardiology	−0.72	0.34	[-1.39, -0.05]	0.034*
Administration	0.48	0.67	[-0.84, 1.8]	0.477
Ambulatory	−0.18	0.11	[-0.4, 0.04]	0.105
Mental health	−0.57	0.46	[-1.46, 0.33]	0.216
Multiple units	−0.36	0.21	[-0.77, 0.04]	0.081
**Shift**				
Day (Ref)				
Evening	0.00	0.11	[-0.23, 0.22]	0.985
Night	−0.28	0.13	[-0.53, -0.03]	0.028*
Rotating shifts	−0.03	0.06	[-0.15, 0.09]	0.609
**Number of hours worked per week**				
<42.5 hours (Ref)				
42.5 hours	0.09	0.07	[-0.05, 0.23]	0.225
>42.5 hours	−0.03	0.06	[-0.15, 0.09]	0.628
**Working years with Syrian refugees**				
1-2 years (Ref)				
3-4 years	−0.01	0.10	[-0.21, 0.19]	0.903
≥5 years	0.06	0.10	[-0.14, 0.25]	0.560
**Self-perceived workload**	0.01	0.00	[0.01, 0.02]	0.000*
**Work stressors**				
Workload stress	0.14	0.04	[0.06, 0.22]	0.001*
Lack of job preparation	0.08	0.04	[0.01, 0.15]	0.035
Job conflict	0.22	0.04	[0.14, 0.29]	0.000*
**Nursing resources**	−0.15	0.05	[-0.24, -0.06]	0.001*
**Nursing resilience**	0.01	0.03	[-0.05, 0.06]	0.840
**Leadership**	−0.06	0.05	[-0.16, 0.03]	0.213
**Teamwork**	−0.10	0.04	[-0.18, -0.02]	0.011*
**General weakness**				
Not at all and a little bit (Ref)				
Strong	0.81	0.07	[0.67, 0.95]	0.000*
**Back pain**				
Not at all and a little bit (Ref)				
Strong	0.37	0.07	[0.24, 0.51]	0.000*
R^2^	0.26			

Nurses working in the intensive care (β = 0.25, p = 0.004), pediatrics/NICU/PICU (β = 0.29, p = 0.002), and emergency units (β = 0.18, p = 0.026) reported significantly higher emotional exhaustion compared to those in medical-surgical units, while those in cardiology had lower exhaustion (β = −0.72, p = 0.034). Working night shifts was also associated with reduced exhaustion compared to day shifts (β = −0.28, p = 0.028).

Work environment variables were strong predictors of self-perceived workload (β = 0.01, p < 0.001), workload stress (β = 0.14, p = 0.001), lack of job preparation (β = 0.08, p = 0.035), and job conflict (β = 0.22, p < 0.001) were all positively associated with emotional exhaustion. In contrast, nursing resources (β = −0.15, p = 0.001) and teamwork (β = −0.10, p = 0.011) were inversely associated. Finally, physical health indicators were among the strongest predictors—nurses reporting strong general weakness (β = 0.81, p < 0.001) or strong back pain (β = 0.37, p < 0.001) exhibited substantially higher emotional exhaustion. The model explained 26% of the variance in emotional exhaustion (R² = 0.26).

#### Country-specific context.

At the country level, several notable differences were observed between Lebanon and Jordan (S3 Table). In Jordan, marital status and work-related factors were most strongly associated with emotional exhaustion. Nurses who were separated, divorced, or widowed reported higher emotional exhaustion compared to those who were single (β = 0.32, p = 0.019). Nurses working in pediatrics/NICU/PICU (β = 0.24, p = 0.025), intensive care (β = 0.25, p = 0.013), and emergency units (β = 0.17, p = 0.049) reported significantly higher emotional exhaustion compared to those in medical-surgical areas. Each one-unit increase in self-perceived workload was associated with a small but significant increase in emotional exhaustion (β = 0.01, p < 0.001), and workload stress (β = 0.12, p = 0.009) and job conflict (β = 0.25, p < 0.001) were also important predictors. Better nursing resources were associated with lower exhaustion (β = −0.11, p = 0.034). Finally, nurses reporting strong back pain (β = 0.33, p < 0.001) or strong general weakness (β = 0.70, p < 0.001) had markedly higher levels of emotional exhaustion.

In Lebanon, psychosocial and work environment factors were more prominent. Nurses working in pediatrics/NICU/PICU units had higher emotional exhaustion compared to those in medical-surgical areas (β = 0.53, p = 0.011). Higher self-perceived workload (β = 0.01, p = 0.002) and workload stress (β = 0.17, p = 0.048) were both positively associated with exhaustion, as was lack of job preparation (β = 0.38, p < 0.001). In contrast, better nursing resources (β = −0.29, p = 0.013) were protective. Similar to Jordan, nurses experiencing strong back pain (β = 0.49, p = 0.002) and strong general weakness (β = 1.15, p < 0.001) reported significantly higher emotional exhaustion.

Overall, emotional exhaustion was shaped by workload, psychosocial stressors, and physical health problems, with strong back pain and general weakness emerging as major contributors across both countries.

### Random forest analysis of key predictors

**Fig 1:**
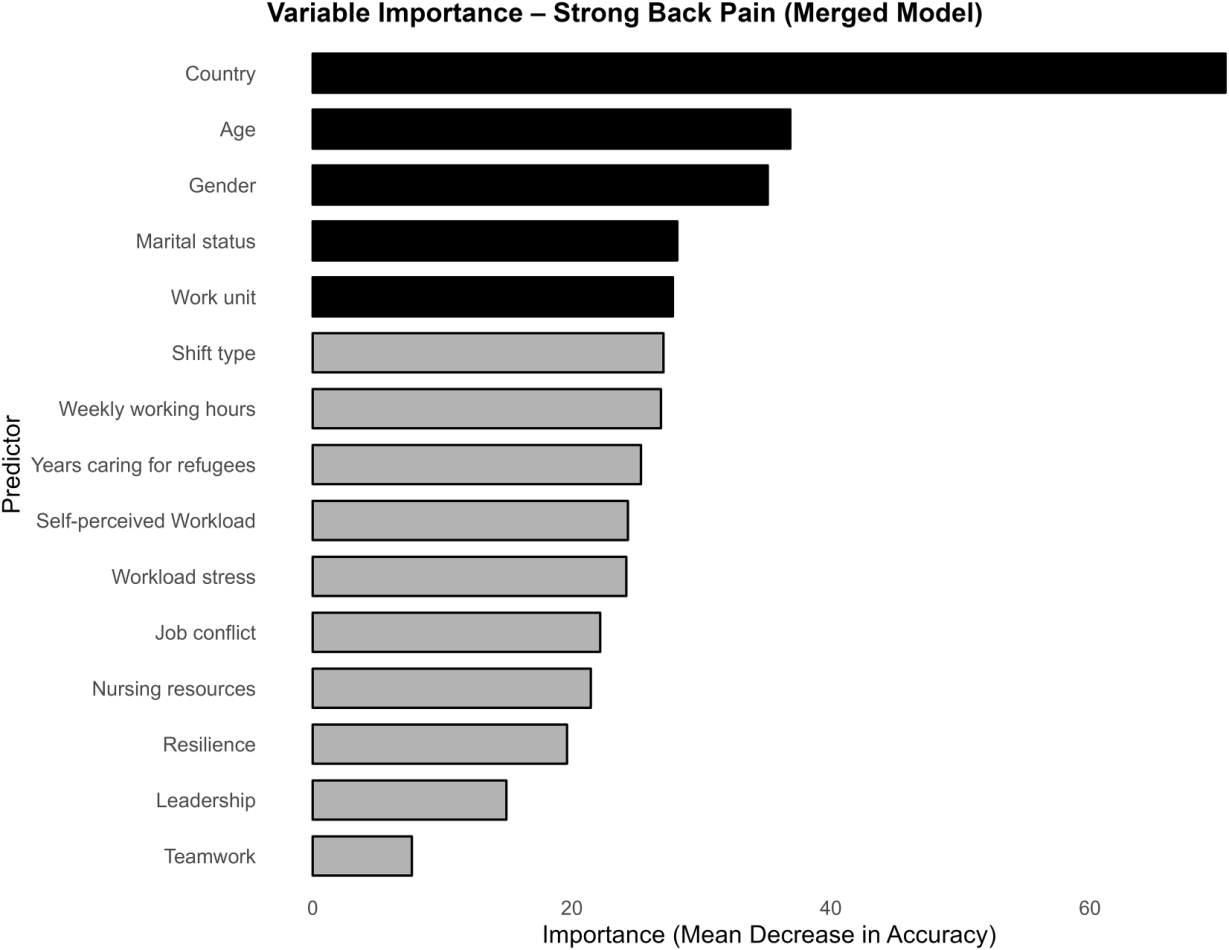
Variable Importance – Strong Back Pain (Merged Model).

For strong back pain (Fig 1, S1–S2 Figs), the merged model highlighted country, age, gender, marital status, and work unit as the variables with the highest relative predictive importance. Country-specific analyses revealed distinct patterns: in Lebanon, work stressors particularly lack of job preparation, job conflict, and self-perceived workload were most important, followed by nursing resources and teamwork; whereas in Jordan, the leading factors were hours worked per week, work unit, lack of job preparation, age, and shift type.

**Fig 2:**
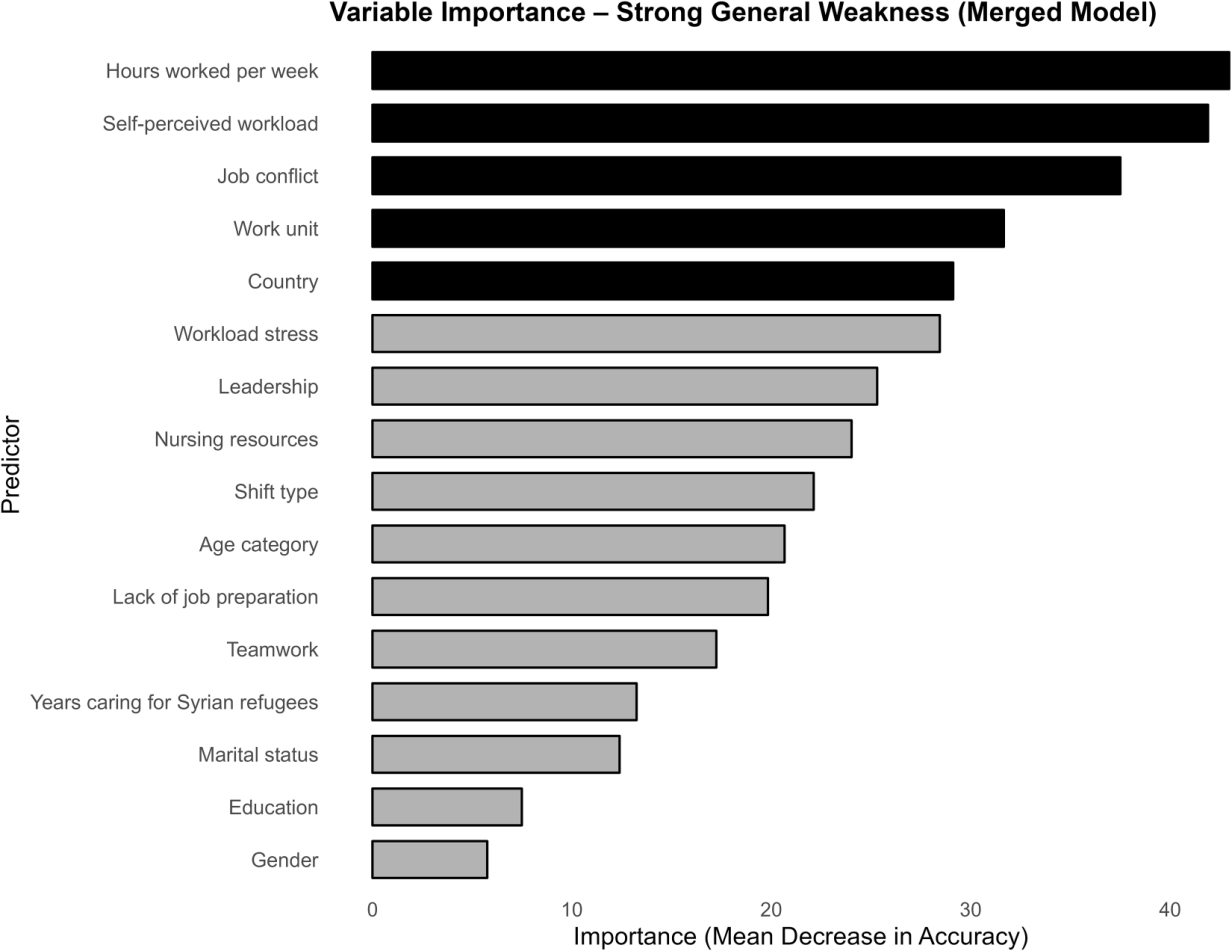
Variable Importance – Strong General Weakness (Merged Model).

In the strong general weakness models (Fig 2, S3–S4 Figs), the merged model identified weekly working hours, self-perceived workload, and job conflict as the strongest predictors, followed by work unit and workload stress. Among Lebanese nurses, job conflict, nursing resources, and teamwork had the highest relative predictive importance. In contrast, in Jordan, hours worked per week, self-perceived workload, and work unit dominated.

**Fig 3:**
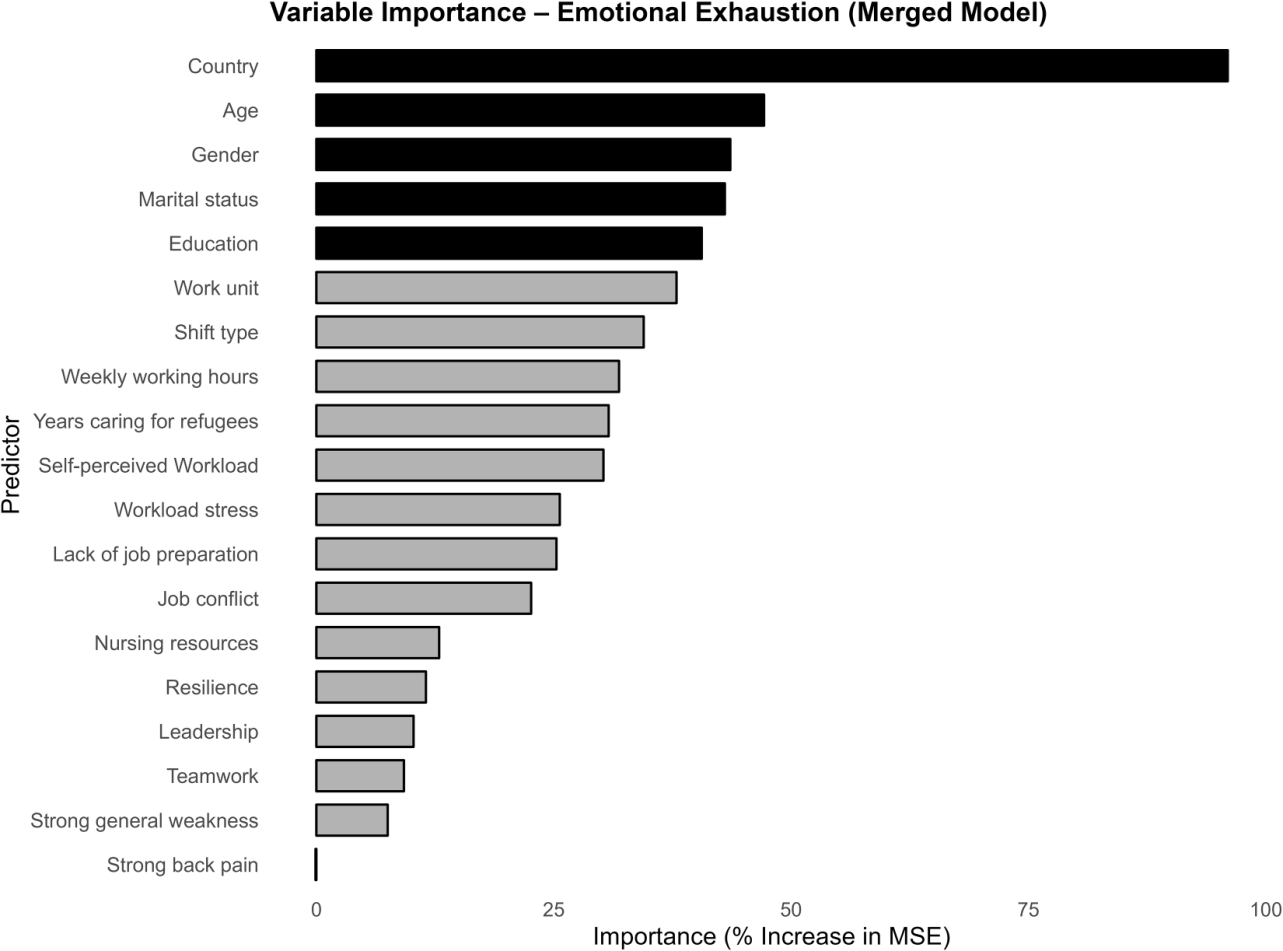
Variable Importance – Emotional Exhaustion (Merged Model).

For emotional exhaustion (Fig 3, S5–S6 Figs), the merged model revealed the importance of country, age, gender, marital status, and education level, with additional contributions from workload-related factors. At the country level, Lebanese nurses’ emotional health was most associated with strong general weakness, strong back pain, lack of job preparation, and nursing resources, whereas in Jordan, the strongest predictors were strong general weakness, strong back pain, nursing resources, work unit and job conflict.

In summary, the random forest analyses confirmed the central role of workload, job conflict, working hours, and physical health problems, while also highlighting country-specific variations in predictor importance.

Figs 1–3 and S1–S6 Figs illustrate the relative feature importance for strong back pain, general weakness, and emotional exhaustion, respectively.

## Discussion

This study aimed to (a) assess nurses’ physical and emotional health in Lebanon and Jordan within the context of the prolonged Syrian refugee crisis, (b) examine and compare the factors associated with these outcomes using merged and country-specific analyses, and (c) identify the key determinants of these outcomes through random forest regression. Overall, the merged models showed that general weakness, back pain, and emotional exhaustion were jointly associated with sociodemographic characteristics (age, gender, marital status), work-related factors (unit type, weekly working hours, shift type), psychosocial work environment indicators (self-perceived workload, workload stress, lack of job preparation, job conflict), and organizational resources (nursing resources and teamwork). Country-specific models then added nuance by highlighting how these determinants manifested differently in Lebanon and Jordan.

These findings align with global calls to strengthen nurses’ protection and retention through safer, better-resourced, and rights-based working conditions. The WHO Global Health explicitly emphasizes preventing harm (including occupational hazards, access to health and mental health services, protection from violence/harassment, and protection from attacks in fragile and conflict-affected settings), alongside inclusivity, adequate resourcing, and safeguarding workers’ rights [15].

### Physical health (general weakness and back pain)

The prevalence of these outcomes was substantial, with nearly six in ten nurses in Lebanon and seven in ten in Jordan reporting strong general weakness, and over 60% in both countries experiencing strong back pain. Although these rates appear high, several factors may explain this pattern. First, the “strong” category reflects self-reported symptom intensity over the past four weeks and may capture a broad range of meaningful discomfort rather than clinically diagnosed conditions. Second, reliance on self-reported measures may introduce reporting bias. Third, these findings must be interpreted within the context of prolonged occupational strain in refugee-serving settings, where sustained workload pressure, staffing constraints, and high-acuity patient care may contribute to elevated symptom reporting. These findings are consistent with the international literature showing that nurses have a high burden of physical complaints and musculoskeletal disorders compared with other professional groups [29,52]. These high rates highlight the significant physical strain experienced by nurses in both settings, highlighting the need for strategies that address musculoskeletal and fatigue-related health risks.

Importantly, global evidence highlight that psychosocial hazards (e.g., chronic stress and burnout) can compound occupational risks by undermining productivity, retention, and care quality; this is particularly concerning in contexts with an ageing nursing workforce, where musculoskeletal injuries may translate into reduced work capacity and fewer hours worked [15].

In our merged models, older age, female gender, longer weekly working hours, and employment in pediatrics/NICU/PICU and obstetrics/gynecology units were associated with greater odds of strong general weakness and/or back pain, while evening and night shifts were associated with somewhat lower odds. Psychosocial factors, including higher self-perceived workload, workload stress, and job conflict, and resilience further increased the odds of poor physical health, whereas better teamwork was protective. These results align with regional and international evidence linking heavy workloads and stressful work environments to musculoskeletal complaints and fatigue among nurses [26,27,53].

Age and gender effects in the merged models align with the literature, but the country-specific data highlight meaningful nuances. Overall, older age was associated with higher odds of back pain, in line with evidence that musculoskeletal strain accumulates as physical strength declines [54,55]. However, in Lebanon, nurses aged ≥41 years reported lower odds of strong general weakness than younger nurses, supporting the integrative review by Alahmadi & Alharbi (2018), which suggested that senior nurses may hold positions with fewer physical demands and reduced night work [30]. In the merged analysis, female gender was a significant predictor of both strong back pain and strong general weakness. However, country-specific models suggested a more nuanced pattern: in Lebanon, female nurses had significantly higher odds of strong back pain and general weakness than males, while in Jordan the association between female gender and strong general weakness was borderline significant. These findings are broadly consistent with descriptive evidence that women report higher levels of physical fatigue and weakness than men [26], which may reflect biological differences, socio-cultural expectations, and gendered role assignments in clinical practice.

Furthermore, in the merged analysis, engaged or married nurses had higher odds of reporting strong general weakness than single nurses, suggesting that combining work and family responsibilities may intensify fatigue. However, this association did not remain significant in country-specific models, likely due to smaller sample sizes and differences in support structures between Lebanon and Jordan.

In the merged analysis, unit emerged as an important determinant of physical health, with nurses working in pediatrics/NICU/PICU and obstetrics/gynecology reporting higher odds of strong general weakness and back pain than those in medical–surgical units. This is partly consistent with previous studies showing that nurses in intensive and pediatric care settings experience greater physical strain and musculoskeletal problems due to high acuity and demanding workloads [26,56]. However, the increased odds observed in obstetrics/gynecology diverge from literature that often reports lower fatigue in this specialty [26], suggesting that in our context factors such as high birth volumes, staffing ratios, and complex case mix, particularly in facilities serving large numbers of refugees, may heighten the physical demands on obstetric nurses. Interestingly, operating-room nurses in the country-specific models had lower odds of strong general weakness and back pain compared with medical–surgical nurses, which contrasts with some evidence of high fatigue among surgical nurses [26] and may reflect the fact that, in our setting, most staff are professional nurses rather than practical nurses and are less involved in manual patient transfers due to the availability of transfer services and more standardized workflows and equipment in the operating room.

Longer weekly working hours were consistently associated with higher odds of both strong back pain and strong general weakness in the merged analysis, indicating that extended schedules represent a key occupational risk factor for nurses in both countries. This association was particularly evident in Jordan, where nurses working 42.5 hours or more per week had significantly higher odds of both outcomes, echoing evidence that extended hours and heavy patient loads increase the risk of low back pain and other health problems [57,58].

In our study, night shifts were associated with lower odds of strong back pain and general weakness compared with day shifts. This pattern is consistent with the slightly lower perceived workload reported on evening shifts, suggesting that reduced workload and fewer high-intensity activities during off-peak hours may lessen the physical strain that contributes to these symptoms.

Psychosocial work environment factors were also central to nurses’ physical health. In the merged models, higher self-perceived workload, workload stress, and job conflict were all associated with increased odds of strong back pain and general weakness. This is consistent with regional and international evidence that excessive physical and psychological workload contributes to musculoskeletal complaints and fatigue among nurses [26,53]. A recent systematic review by AlAmmari et al. (2025) similarly highlighted high physical workload as a key risk factor for low back pain among hospital-based healthcare professionals [27], reinforcing the detrimental impact of workload-related stressors on nurses’ physical health in Middle Eastern contexts. The positive association between resilience and back pain may appear counterintuitive, but it may reflect the way resilience operates in highly demanding work environments. In fragile health systems, nurses with greater resilience may remain in their posts, continue to cope actively with heavy workloads, and tolerate discomfort for longer periods, which may expose them to sustained physical strain and “dysfunctional persistence” rather than symptom relief. The pain literature also suggests that resilience is not a simple linear protective factor; instead, it may coexist with continued activity despite pain, especially when workers are motivated to maintain functioning under pressure [59]. Country-specific analyses suggested that in Jordan, workload intensity and workload stress were particularly pronounced, whereas in Lebanon, lack of job preparation and job conflict were more prominent, echoing evidence that insufficient training and conflictual work environments worsen nurses’ health and turnover [60,61]. Conversely, in Lebanon, higher nursing resources were associated with lower odds of both strong back pain and general weakness, and better teamwork was additionally protective for general weakness, supporting prior work that social support and supportive organizational climates mitigate fatigue and strain among nurses [30].

### Emotional health (emotional exhaustion)

In the merged analysis, emotional exhaustion was strongly associated with both work-related and health-related variables. Jordanian nurses, on average, reported higher emotional exhaustion than Lebanese nurses, mirroring findings from Gulf settings where high emotional exhaustion levels have been linked to staff shortages and heavy workloads [62].

Nurses aged 31–40 reported lower emotional exhaustion than those below 30 years, suggesting that early-career nurses may be more vulnerable to burnout. Younger nurses often face steeper learning curves and less-developed coping strategies, whereas mid-career nurses may benefit from greater experience and support at work [30]. However, this pattern did not reach significance in the country-specific analyses, likely reflecting reduced power after stratification. In addition, in the merged model, nurses who were separated, divorced, or widowed reported higher emotional exhaustion than single nurses, suggesting that family circumstances may heighten vulnerability to exhaustion. This pattern was also evident in Jordan and is consistent with evidence that strained or complex family roles can intensify the challenge of balancing work and personal life [63]. From a policy perspective, this “early-career vulnerability” is concerning because recent global nursing-sector assessments suggest that many countries still lack formal supports that could buffer stress and prevent attrition, including structured support for new graduates and access to workplace psychological/mental health support [64].

Work-related and psychosocial work environment factors were strongly linked to emotional exhaustion. In the merged model, nurses working night shifts reported lower exhaustion than those on day shifts, which is consistent with our descriptive data showing slightly lower workload scores on night shifts suggesting that reduced workload likely underpins this effect. In contrast, higher self-perceived workload, workload stress, lack of job preparation, and job conflict were all associated with increased emotional exhaustion, in line with evidence that demanding, poorly supported roles and interpersonal tensions drive burnout in healthcare workers [55]. Conversely, greater nursing resources and better teamwork were consistently protective, supporting literature that emphasizes the buffering role of adequate staffing, supportive leadership, and collegial relationships [30]. Country-specific analyses reflected a picture of lack of job preparation particularly prominent in Lebanon, whereas job conflict was more prominent in Jordan, indicating that although the overall pattern of “high demands and low support” contributing to exhaustion is shared, the specific stressors that dominate may differ between the two settings.

Unit of work was also associated with emotional exhaustion. In the merged model, nurses in intensive care, pediatrics/NICU/PICU, and emergency units reported higher exhaustion than those in medical–surgical units, whereas cardiology nurses reported lower exhaustion. The elevated exhaustion in high-acuity units is consistent with prior studies showing that intensive and emergency care nurses face higher physical and emotional demands, leading to greater fatigue and burnout [26,65,66]. Country-specific analyses echoed this pattern, particularly in Jordan, where ICU, pediatrics/NICU/PICU, and emergency units were all associated with higher emotional exhaustion, suggesting that the concentration of critically ill and complex patients in these units is a key driver of nurses’ emotional strain.

Finally, our both merged, and country-specific results revealed that nurses experiencing strong back pain or general weakness were also more likely to report higher emotional exhaustion. This highlights the interplay between physical and emotional health, consistent with evidence that musculoskeletal strain and psychosocial stressors often act synergistically to intensify fatigue and burnout [27].

This interdependence further supports investing in structured mental well-being supports as part of workforce protection. However, global reporting suggests that formal “mental well-being care packages” for health workers are not universal, with fewer than half of surveyed countries reporting their availability. This gap is important for Lebanon and Jordan given the compounded pressures of workload, conflict-related stressors, and refugee-responsive service delivery [15].

### Insights from random forest analysis

The random forest models directly addressed our third objective by identifying the most influential predictors across outcomes and countries. Looking at the results, the importance profiles for back pain and general weakness point to slightly different mechanisms. For strong back pain, country was by far the most influential predictor, followed by core sociodemographic and structural factors—age, gender, marital status, and work unit—with work schedule, years caring for refugees, and workload- or conflict-related variables playing a secondary role. This suggests that cross-country differences and basic workforce composition may contribute to differences in predicted back pain risk. By contrast, for strong general weakness, the top predictors were hours worked per week, self-perceived workload, job conflict, work unit, and overall workload, with country and sociodemographic factors contributing less. This pattern is consistent with the possibility that general weakness reflects cumulative response to intensive and stressful work environments, though longitudinal studies are needed to confirm this interpretation.

For emotional exhaustion, the random forest model showed that contextual and individual characteristics particularly country, age, gender, marital status, and education level, ranked highest in relative predictive importance, while work unit, shift type, and weekly working hours formed a second tier of importance. Psychosocial work factors such as self-perceived workload, workload stress, lack of job preparation, job conflict, and organizational resources made smaller but still meaningful contributions, indicating their additional predictive relevance beyond broader structural and personal characteristics. This pattern suggests that, in the pooled data, sociodemographic and contextual characteristics exhibited higher relative predictive importance than certain day-to-day work environment variables for emotional exhaustion, without implying causal determination.

This study provides important insights into the physical and emotional health of nurses working with refugees in Lebanon and Jordan, two countries at the center of one of the region’s most severe humanitarian crises. These are important observations reflected in other studies in conflict zones and refugee settings [31,67].

In refugee settings and protracted conflict-adjacent displacement contexts, nurses’ workload increases often in volume, acuity, and is consistently associated with worse physical and emotional outcomes. Increased exposure to family separation, violence, and suffering of the vulnerable increases the risk of secondary traumatic stress. This emphasizes the need for supportive strategies and consideration of models that allow surge staffing and provision of social support.

### Implications for practice and policy

These findings have important policy and practice implications for Lebanon and Jordan. Priorities should include workload regulation, safer staffing levels or nurse-to-patient ratios, targeted training and preparation for high-demand units, and stronger managerial, peer, and organizational support. Because both countries are working within constrained health systems and ongoing workforce shortages, these interventions should be realistic, context-sensitive, and focused on the units and shifts with the heaviest burden.

### Strengths and limitations

A significant strength is its large, binational sample and the use of validated instruments, which improve the robustness and comparability of findings. The explicit cross-country design and the use of both combined and country-specific models allowed us to identify common determinants while also capturing contextual differences between Lebanon and Jordan. Furthermore, combining regression and random forest approaches improves the study by determining not just relationships but also the relative relevance of key factors.

However, this study comes with limitations. First, the study’s cross-sectional design limits the causal interpretation of the observed associations. In particular, reverse causality between physical health and emotional exhaustion cannot be ruled out, as physical symptoms may contribute to emotional exhaustion while emotional exhaustion may also influence the reporting or experience of physical symptoms.

Second, relying on self-reported data might lead to reporting bias, especially for sensitive outcomes such as emotional exhaustion or physical health. Third, the exclusion of academic and supervisory nurses may limit the generalizability of the findings to the larger nursing workforce. Finally, while machine learning techniques have provided important insights into variable relevance, they do not explain causal pathways and may be affected by data format and model parameters.

## Conclusion

This study shows that hospital nurses caring for Syrian refugees in Lebanon and Jordan carry a substantial burden of poor health, with high rates of strong back pain, general weakness, and emotional exhaustion. Using merged and country-specific regression and random forest models, we found that these outcomes are jointly associated with sociodemographic factors (age, gender, and marital status), occupational features (unit type, shift, weekly working hours), and psychosocial work conditions (self-perceived workload, workload stress, lack of job preparation, job conflict), while nursing resources and teamwork consistently emerged as protective. Long working hours and high-acuity units were particularly important for physical health, whereas emotional exhaustion was further associated with family circumstances and country context, and was strongly linked to physical symptoms. Together, these findings highlight the need for integrated interventions that reduce excessive workload and conflict, optimize staffing and preparation, especially in high-acuity, refugee-serving units, and strengthen supportive team and leadership climates to protect nurse well-being and sustain health system resilience in protracted crisis settings. These findings may also guide workforce policies in other refugee-hosting countries facing comparable health system strain.

## Supporting information

S1 Table Adjusted multiple logistic regression analyses for general weakness outcome. (DOCX)

S2 Table Adjusted multiple logistic regression analyses for back pain outcome. (DOCX)

S3 Table Linear regression models for emotional health (emotional exhaustion) outcome. (DOCX)

S1 Fig Variable Importance – Strong Back Pain (Lebanon). (TIF)

S2 Fig Variable Importance – Strong Back Pain (Jordan). (TIF)

S3 Fig Variable Importance – Strong General Weakness (Lebanon). (TIF)

S4 Fig Variable Importance – Strong General Weakness (Jordan). (TIF)

S5 Fig Variable Importance – Emotional Exhaustion (Lebanon). (TIF)

S6 Fig Variable Importance – Emotional Exhaustion (Jordan). (TIF)

S1 Checklist Inclusivity in global research. (DOCX)
