# Supplementary material for: Physical and emotional health among nurses in protracted crisis settings in Lebanon and Jordan: A cross-sectional study
Source: PLoS One. 2026 Jun 23;21(6):e0352022. doi: 10.1371/journal.pone.0352022 (PMC13289918; doi:10.1371/journal.pone.0352022)
Supplement: S1 Table — (DOCX) [file pone.0352022.s001.docx]

**S1 Table.** **Adjusted multiple logistic regression analyses for general weakness outcome.**

|  | **Lebanon** | | | **Jordan** | | |
| --- | --- | --- | --- | --- | --- | --- |
|  | **A-OR** | **95% CI** | **P-value** | **A-OR** | **95% CI** | **P-value** |
| **Age (years)** |  |  |  |  |  |  |
| 20- 30 (Ref) | - | - | **-** | - | - | - |
| 31- 40 | 0.99 | 0.62-1.56 | 0.956 | 1.25 | 0.94-1.65 | 0.126 |
| ≥41 | 0.49 | 0.25-0.93 | **0.029** | 1.48 | 0.95-2.33 | 0.086 |
| **Gender** |  |  |  |  |  |  |
| Male (Ref) | - | - | - | - | - | - |
| Female | 1.80 | 1.17-2.77 | **0.008** | 1.26 | 1.00-1.59 | **0.052** |
| **Marital status** |  |  |  |  |  |  |
| Single (Ref) | - | - | - | - | - | - |
| Engaged/married | 1.14 | 0.75-1.74 | 0.535 | 1.26 | 0.93-1.69 | 0.131 |
| Separated/divorced/widowed | 1.89 | 0.56-6.33 | 0.303 | 1.40 | 0.85-2.30 | 0.187 |
| **Work unit** |  |  |  |  |  |  |
| Medical-surgical area/burns (Ref) | - | - | - | - | - | - |
| Intensive care unit | - | - | - | 1.16 | 0.79-1.71 | 0.450 |
| Pediatrics/NICU/PICU | - | - | - | 1.56 | 1.06-2.28 | **0.023** |
| Obstetrics/gynecology | - | - | - | 1.50 | 0.99-2.26 | 0.055 |
| Renal dialysis unit | - | - | - | 0.73 | 0.39-1.38 | 0.330 |
| Operating room | - | - | - | 0.64 | 0.41-0.98 | **0.040** |
| Emergency | - | - | - | 1.31 | 0.95-1.80 | 0.099 |
| Ambulatory | - | - | - | 0.70 | 0.78-1.04 | 0.074 |
| **Number of hours worked per week** |  |  |  |  |  |  |
| <42.5 hours (Ref) | - | - | - | - | - | - |
| 42.5 hours | 0.72 | 0.43-1.21 | 0.216 | 1.74 | 1.32-2.29 | **<0.001** |
| >42.5 hours | 0.78 | 0.49-1.26 | 0.310 | 2.26 | 1.74-2.95 | **<0.001** |
| **Shift** |  |  |  |  |  |  |
| Day (Ref) | - | - | - | - | - | - |
| Evening | - | - | - | 0.82 | 0.51-1.33 | 0.425 |
| Night | - | - | - | 0.58 | 0.32-1.03 | 0.063 |
| Rotating shifts | - | - | - | 0.84 | 0.65-1.09 | 0.187 |
| **Working years with Syrian refugees** |  |  |  |  |  |  |
| 1-2 years (Ref) | - | - | - | - | - | - |
| 3-4 years | 1.06 | 0.63-1.77 | 0.830 | 1.04 | 0.65-1.65 | 0.871 |
| ≥5 years | 1.39 | 0.83-2.33 | 0.212 | 1.25 | 0.79-1.96 | 0.339 |
| **Self-perceived workload** | 1.01 | 1.00-1.02 | 0.061 | 1.02 | 1.01-1.03 | **<0.001** |
| **Work stressors** |  |  |  |  |  |  |
| Workload stress | 1.11 | 0.84-1.45 | 0.466 | 1.17 | 1.00-1.38 | 0.055 |
| Lack of job preparation | 1.20 | 0.89-1.61 | 0.235 | 0.91 | 0.79-1.06 | 0.234 |
| Job conflict | 1.57 | 1.18-2.08 | **0.002** | 1.26 | 1.08-1.46 | **0.003** |
| **Nursing resources** | 0.62 | 0.44-0.89 | **0.009** | **-** | **-** | **-** |
| **Nursing resilience** | 1.02 | 0.82-1.26 | 0.876 | 1.09 | 0.98-1.20 | 0.114 |
| **Leadership** | 0.90 | 0.62-1.31 | 0.591 | **-** | **-** | **-** |
| **Teamwork** | 0.63 | 0.44-0.90 | **0.011** | **-** | **-** | **-** |
