# Supplementary material for: Physical and emotional health among nurses in protracted crisis settings in Lebanon and Jordan: A cross-sectional study
Source: PLoS One. 2026 Jun 23;21(6):e0352022. doi: 10.1371/journal.pone.0352022 (PMC13289918; doi:10.1371/journal.pone.0352022)
Supplement: S2 Table — (DOCX) [file pone.0352022.s002.docx]

**S2 Table. Adjusted multiple logistic regression analyses for back pain outcome.**

|  | **Lebanon** | | | **Jordan** | | |
| --- | --- | --- | --- | --- | --- | --- |
|  | **A-OR** | **95% CI** | **P-value** | **A-OR** | **95% CI** | **P-value** |
| **Age (years)** |  |  |  |  |  |  |
| 20- 30 (Ref) | - | - | **-** | - | - | - |
| 31- 40 | 0.76 | 0.48-1.20 | 0.239 | 1.58 | 1.21-2.06 | **0.001** |
| ≥41 | 0.66 | 0.35-1.26 | 0.212 | 3.05 | 1.95-4.79 | **<0.001** |
| **Gender** |  |  |  |  |  |  |
| Male (Ref) | **-** | **-** | **-** | **-** | **-** | **-** |
| Female | 1.81 | 1.18-2.76 | **0.006** | 1.17 | 0.94-1.46 | 0.168 |
| **Marital status** |  |  |  |  |  |  |
| Single (Ref) | - | - | - | **-** | **-** | **-** |
| Engaged/married | 1.24 | 0.82-1.88 | 0.309 | 1.12 | 0.84-1.48 | 0.446 |
| Separated/divorced/widowed | 0.81 | 0.26-2.45 | 0.703 | 1.22 | 0.76-1.97 | 0.406 |
| **Work unit** |  |  |  |  |  |  |
| Medical-surgical area/burns (Ref) | - | - | - | **-** | **-** | **-** |
| Intensive care unit | - | - | - | 0.70 | 0.49-1.01 | 0.054 |
| Pediatrics/NICU/PICU | - | - | - | 1.58 | 1.11-2.26 | **0.011** |
| Obstetrics/gynecology | - | - | - | 1.53 | 1.04-2.24 | **0.031** |
| Renal dialysis unit | - | - | - | 0.62 | 0.33-1.16 | 0.132 |
| Operating room | - | - | - | 0.60 | 0.39-0.91 | **0.018** |
| Emergency | - | - | - | 1.20 | 0.89-1.62 | 0.229 |
| Ambulatory |  |  |  | 0.88 | 0.60-1.31 | 0.540 |
| **Education** |  |  |  |  |  |  |
| Technical nursing diploma (BT, TS, LT) (Ref) | - | - | - | **-** | **-** | **-** |
| University degree | - | - | - | 0.90 | 0.66-1.23 | 0.496 |
| **Number of hours worked per week** |  |  |  |  |  |  |
| <42.5 hours (Ref) | - | - | - | - | - | - |
| 42.5 hours | 0.65 | 0.39-1.07 | 0.092 | 1.95 | 1.50-2.53 | **<0.001** |
| >42.5 hours | 1.01 | 0.63-1.63 | 0.973 | 3.00 | 2.34-3.85 | **<0.001** |
| **Shift** |  |  |  |  |  |  |
| Day (Ref) | - | - | - | **-** | **-** | **-** |
| Evening | 0.91 | 0.40-2.07 | 0.824 | 0.64 | 0.41-1.02 | 0.060 |
| Night | 0.74 | 0.34-1.61 | 0.449 | 0.63 | 0.36-1.10 | 0.105 |
| Rotating shifts | 1.11 | 0.73-1.70 | 0.624 | 0.88 | 0.69-1.13 | 0.315 |
| **Working years with Syrian refugees** |  |  |  |  |  |  |
| 1-2 years (Ref) | - | - | - | **-** | **-** | **-** |
| 3-4 years | 1.11 | 0.67-1.86 | 0.683 | 1.14 | 0.74-1.77 | 0.544 |
| ≥5 years | 1.55 | 0.93-2.60 | 0.096 | 1.48 | 0.97-2.26 | 0.072 |
| **Self-perceived workload** | 1.01 | 1.00-1.02 | 0.086 | 1.01 | 1.00-1.02 | **0.022** |
| **Work stressors** |  |  |  |  |  |  |
| Workload stress | 1.00 | 0.77-1.30 | 0.980 | 1.21 | 1.04-1.40 | **0.016** |
| Lack of job preparation | 1.46 | 1.09-1.97 | **0.012** | 1.00 | 0.87-1.14 | 0.998 |
| Job conflict | 1.37 | 1.04-1.80 | **0.025** | **-** | **-** | **-** |
| **Nursing resources** | 0.64 | 0.45-0.90 | **0.011** | 1.16 | 1.00-1.35 | 0.052 |
| **Leadership** | 1.05 | 0.74-1.51 | 0.777 | **-** | **-** | **-** |
| **Teamwork** | 0.81 | 0.57-1.14 | 0.218 | **-** | **-** | **-** |
| **Resilience** | 1.06 | 0.86-1.31 | 0.559 |  |  |  |
