# Supplementary material for: Physical and emotional health among nurses in protracted crisis settings in Lebanon and Jordan: A cross-sectional study
Source: PLoS One. 2026 Jun 23;21(6):e0352022. doi: 10.1371/journal.pone.0352022 (PMC13289918; doi:10.1371/journal.pone.0352022)
Supplement: S3 Table — (DOCX) [file pone.0352022.s003.docx]

**S3 Table.** **Linear regression models for emotional health (emotional exhaustion) outcome.**

|  | **Lebanon**  (N=976) | | | **Jordan**  (N=2012) | | |
| --- | --- | --- | --- | --- | --- | --- |
| **Mean (SD)** | 3.49 (1.70) | | | 4.13 (1.39) | | |
|  | **β** | **S.E.** | **P-value** | **β** | **S.E.** | **P-value** |
| **Age (years)** |  |  |  |  |  |  |
| 20- 30 (Ref) | **-** | **-** | **-** | **-** | **-** | **-** |
| 31- 40 | -0.15 | 0.15 | 0.321 | -0.14 | 0.07 | 0.067 |
| ≥41 | -0.27 | 0.21 | 0.199 | -0.01 | 0.12 | 0.939 |
| **Gender** |  |  |  |  |  |  |
| Male (Ref) | **-** | **-** | **-** | **-** | **-** | **-** |
| Female | -0.24 | 0.15 | 0.106 | -0.04 | 0.07 | 0.588 |
| **Marital status** |  |  |  |  |  |  |
| Single (Ref) | **-** | **-** | **-** | **-** | **-** | **-** |
| Engaged/married | -0.04 | 0.13 | 0.774 | 0.10 | 0.08 | 0.224 |
| Separated/divorced/widowed | 0.08 | 0.33 | 0.818 | 0.32 | 0.14 | **0.019** |
| **Work unit** |  |  |  |  |  |  |
| Medical-surgical area/burns (Ref) | **-** | **-** | **-** | **-** | **-** | **-** |
| Intensive care unit | 0.33 | 0.2 | 0.097 | 0.25 | 0.10 | **0.013** |
| Pediatrics/NICU/PICU | 0.53 | 0.21 | **0.011** | 0.24 | 0.11 | **0.025** |
| Obstetrics/gynecology | 0.18 | 0.25 | 0.480 | 0.16 | 0.10 | 0.131 |
| Renal dialysis unit | 0.45 | 0.3 | 0.138 | 0.08 | 0.18 | 0.646 |
| Operating room | 0.52 | 0.28 | 0.062 | -0.15 | 0.15 | 0.326 |
| Emergency | 0.41 | 0.22 | 0.060 | 0.17 | 0.08 | **0.049** |
| Oncology | 0.52 | 0.35 | 0.143 | - | - | - |
| Cardiology | -0.43 | 0.37 | 0.247 | - | - | - |
| Administration | 0.7 | 0.68 | 0.303 | - | - | - |
| Ambulatory | -0.29 | 0.49 | 0.557 | -0.22 | 0.12 | 0.059 |
| Mental health | -0.2 | 0.45 | 0.660 | - | - | - |
| Multiple units | -0.1 | 0.22 | 0.636 | - | - | - |
| **Education** |  |  |  |  |  |  |
| Technical nursing diploma (BT, TS, LT) (Ref) | **-** | **-** | **-** | **-** | **-** | **-** |
| University degree | -0.22 | 0.13 | 0.095 | 0.04 | 0.09 | 0.688 |
| **Number of hours worked per week** |  |  |  |  |  |  |
| <42.5 hours (Ref) | 0.17 | 0.16 | 0.304 | 0.11 | 0.08 | 0.193 |
| 42.5 hours | -0.05 | 0.15 | 0.721 | -0.04 | 0.07 | 0.599 |
| >42.5 hours | **-** | **-** | **-** | **-** | **-** | **-** |
| **Shift** |  |  |  |  |  |  |
| Day (Ref) | **-** | **-** | **-** | **-** | **-** | **-** |
| Evening | 0.01 | 0.35 | 0.977 | 0.00 | 0.11 | 1.000 |
| Night | -0.43 | 0.23 | 0.062 | -0.17 | 0.15 | 0.260 |
| Rotating shifts | -0.21 | 0.14 | 0.132 | -0.02 | 0.07 | 0.830 |
| **Working years with Syrian refugees** |  |  |  |  |  |  |
| 1-2 years (Ref) | - | - | - | - | - | - |
| 3-4 years | 0.07 | 0.18 | 0.696 | 0.01 | 0.14 | 0.946 |
| ≥5 years | 0.05 | 0.16 | 0.770 | 0.09 | 0.13 | 0.471 |
| **Self-perceived workload** | 0.01 | 0.00 | **0.002** | 0.01 | 0.00 | **<0.001** |
| **Work stressors** |  |  |  |  |  |  |
| Workload stress | 0.17 | 0.09 | **0.048** | 0.12 | 0.05 | **0.009** |
| Lack of job preparation | 0.38 | 0.09 | **<0.001** | 0.00 | 0.04 | 0.911 |
| Job conflict | -0.02 | 0.09 | 0.808 | 0.25 | 0.04 | **<0.001** |
| **Nursing resources** | -0.29 | 0.12 | **0.013** | -0.11 | 0.05 | **0.034** |
| **Nursing resilience** | -0.02 | 0.08 | 0.823 | 0.03 | 0.03 | 0.420 |
| **Leadership** | -0.06 | 0.1 | 0.540 | -0.06 | 0.06 | 0.281 |
| **Teamwork** | -0.15 | 0.11 | 0.179 | -0.07 | 0.04 | 0.090 |
| **Physical Health (general weakness)** |  |  |  |  |  |  |
| Not at all and a little bit (Ref) | - | - | - | - | - | - |
| Strongly | 1.15 | 0.17 | **<0.001** | 0.70 | 0.08 | **<0.001** |
| **Physical Health (back pain)** |  |  |  |  |  |  |
| Not at all and a little bit (Ref) | - | - | - | - | - | - |
| Strongly | 0.49 | 0.16 | **0.002** | 0.33 | 0.08 | **<0.001** |
| R^2^ | 0.432 | | | 0.193 | | |
